# Metabolic and Structural Alterations in the Motor System Following Spinal Cord Injury: An In‐Vivo ^1^H‐MR Spectroscopy Investigation

**DOI:** 10.1002/jnr.70071

**Published:** 2025-07-24

**Authors:** Simon Schading‐Sassenhausen, Anna Lebret, Kadir Şimşek, Pauline Gut, Sabrina Imhof, Björn Zörner, Roland Kreis, Patrick Freund, Maryam Seif

**Affiliations:** ^1^ Spinal Cord Injury Center, Balgrist University Hospital University of Zurich Zurich Switzerland; ^2^ Cardiff University Brain Research Imaging Centre (CUBRIC) Cardiff University Cardiff UK; ^3^ School of Computer Science and Informatics Cardiff University Cardiff UK; ^4^ Department of Diagnostic and Interventional Radiology Lausanne University Hospital and University of Lausanne Lausanne Switzerland; ^5^ Swiss Paraplegic Centre Nottwil Switzerland; ^6^ Magnetic Resonance Methodology, Institute of Diagnostic and Interventional Neuroradiology University of Bern Bern Switzerland; ^7^ Translational Imaging Center, Swiss Institute for Translational and Entrepreneurial Medicine Bern Switzerland; ^8^ Institute of Psychology University of Bern Bern Switzerland; ^9^ Department of Imaging Neuroscience, UCL Queen Square Institute of Neurology University College London London UK; ^10^ Department of Neurophysics Max Planck Institute for Human Cognitive and Brain Sciences Leipzig Germany

**Keywords:** ^1^H‐MR spectroscopy, lumbar cord, motor dysfunction, spinal cord injury

## Abstract

Spinal cord injury (SCI) disrupts spinal tracts and neuronal pathways, including those in the primary motor cortex (M1) and the lumbar cord enlargement (LCE) involved in motor control. This study sought to determine whether metabolite concentrations deviate between SCI and healthy controls (HC) in M1 and LCE using proton magnetic resonance spectroscopy (^1^H‐MRS) and structural MRI, and if these correlate with clinical impairment. Sixteen chronic SCI (mean age: 54.7 ± 14.8y) and 19 HCs (mean age: 53.2 ± 18.8y) underwent ^1^H‐MRS to quantify metabolites along with T_1_‐ and T_2_*‐weighted MRI to assess tissue structural changes. Associations between metabolic and structural changes and clinical impairment were also assessed. Patients showed significant atrophy in both white matter of the LCE (HC: 37.7 ± 4.7 mm^2^, SCI: 33.9 ± 3.7 mm^2^, Δ = −10.1%, *p* = 0.015) and gray matter (HC: 20.9 ± 2.1 mm^2^, SCI: 19.4 ± 1.5 mm^2^, Δ = −7.2%, *p* = 0.022). Total N‐acetylaspartate (tNAA) with respect to total creatine (tCr) was reduced in M1 of SCI (HC: 1.94 ± 0.21, SCI: 1.77 ± 0.14, ∆ = −8.8%, *p* = 0.006) and in the LCE (HC: 2.48 ± 0.76, SCI: 1.81 ± 0.80, ∆ = −27.0%, *p* = 0.02). In conclusion, reduced tNAA/tCr in both the atrophied LCE and M1 suggests widespread neuronal changes including cell atrophy and/or cell loss after injury. These findings provide in vivo evidence for retrograde and trans‐synaptic neurodegeneration, which may underline the atrophy observed in the motor system in SCI. Ultimately, this highlights the potential for metabolic and structural biomarkers to improve the monitoring of subtle neurodegeneration following SCI and to enhance future regenerative treatment strategies.

## Graphical abstract

Proton magnetic resonance spectroscopy (MRS) revealed injury‐induced changes in neuronal metabolites in the primary motor cortex and lumbar cord enlargement of patients with spinal cord injury.

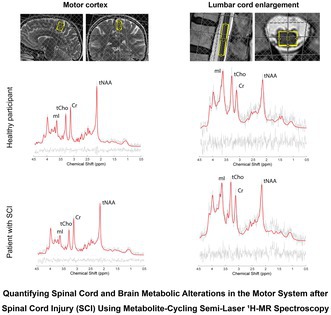

**Summary**

- Spinal cord injury (SCI) affects not only the site of lesion but also triggers widespread changes across the entire nervous system.
- This study applied advanced MR imaging and spectroscopy methods in the brain's motor cortex and lumbar cord enlargement to reveal injury‐induced structural and metabolic alterations.
- These findings provide in vivo evidence of remote neuronal damage across the motor system following SCI.
- A deeper understanding of these changes may inform the development of future treatments for SCI or other neurological disorders.

## 1 Introduction

Spinal cord injury (SCI) leads to immediate damage at the focal injury site and triggers a secondary injury series, such as inflammation, neuronal and glial cell death, causing retrograde, anterograde, and trans‐synaptic neurodegeneration (Ahuja et al. 2017). This is followed by changes in macro‐ and micro‐structural architecture rostro‐caudal to the injury in the spinal cord and the brain (David, Mohammadi, et al. 2019; Hill 2017; Kalil and Schneider 1975). The lumbar cord enlargement (LCE), which is responsible for the main sensorimotor innervation of the lower extremities, is affected by significant degenerative processes including demyelination and atrophy after SCI (David, Seif, et al. 2019; David et al. 2022). The secondary motoneurons within the LCE receive direct input from primary motoneurons located in the leg area of the primary motor cortex (M1) (Lacroix et al. 2004). However, while structural changes in the LCE have been described (David, Seif, et al. 2019), the metabolic alterations that may contribute to the underlying mechanisms of tissue atrophy after SCI are yet understudied. Furthermore, studying the implications of metabolic alterations for functional recovery of the lower limbs, and the association with supraspinal metabolic changes, could provide insight into the system‐wide impact of SCI on motor function and recovery potential.

Proton magnetic resonance spectroscopy (^1^H‐MRS) is a valuable quantitative tool sensitive to chemical components of the tissue and related pathophysiological changes for various pathologies including epilepsy, multiple sclerosis, stroke, metabolic diseases (Oz et al. 2014; Ross and Bluml 2001) and SCI (Liu et al. 2023; Pfyffer et al. 2023; Wyss et al. 2019). The most interesting metabolites in SCI are total N‐acetylaspartate (tNAA), which is a marker of neuronal density and viability, total choline (tCho), a marker of cell membrane density and integrity, and myo‐inositol (mI), which reflects glial density and integrity and therefore is elevated in the setting of gliosis (Soares and Law 2009). In the cervical cord, decreases of tNAA and tCho with respect to mI have been detected, demonstrating the microstructural consequences of SCI remote from the lesion (Wyss et al. 2019).

In this study, we applied single‐voxel MR spectroscopy (MRS) using a semi‐Localized by Adiabatic Selective Refocusing (LASER) technique with metabolite cycling (Dreher and Leibfritz 2005; Döring et al. 2018) to measure metabolic changes across the motor system in SCI. The semi‐LASER technique is robust against B_1_‐field inhomogeneities, especially in challenging areas like the spinal cord (Scheenen et al. 2007). Measurements were conducted back‐to‐back on the same day in both the brain motor cortex and the LCE. We hypothesized that (i) metabolic markers of neuronal integrity and membrane turnover are reduced in atrophied areas in SCI while markers of gliosis are increased, (ii) the magnitude of the metabolic changes is related to clinical impairments, (iii) the changes observed in the LCE are associated with those in M1, and (iv) both the LCE and M1 show atrophy.

## 2 Methods

### 2.1 Standard Protocol Approvals, Registrations, and Study Population

The study protocol complied with the Declaration of Helsinki and was approved by the Kantonale Ethikkommission Zürich (EK‐2018‐00,937 and EK‐2020‐00,247). Written informed consent was obtained from all participants. Sixteen chronic SCI patients (age [mean ± SD]: 54.7 ± 14.8 years, male (m)/female (f): 14/2) and 19 healthy controls (HC) (age [mean ± SD]: 53.2 ± 18.8 years, m/f: 11/8) were recruited between March 2021 and February 2024 at the Balgrist University Hospital. Participants were aged 18–75 years with no concomitant head or brain lesions. Inclusion criteria for patients included an injury affecting the cervical cord or thoracic cord. Exclusion criteria included pre‐existing neurological disorders, pregnancy, MRI contraindication, lumbar lesions, or implants causing metal artifacts within the MRS voxel region of interest.

### 2.2 Clinical Assessment

SCI patients underwent a comprehensive International Standards for Neurological Classification of Spinal Cord Injury (ISNCSCI)‐based clinical examination for the assessment of motor, light touch, and pin‐prick score (Rupp et al. 2021) during their routine check‐up.

### 2.3 MRI And MRS Acquisition

**FIGURE 1:**
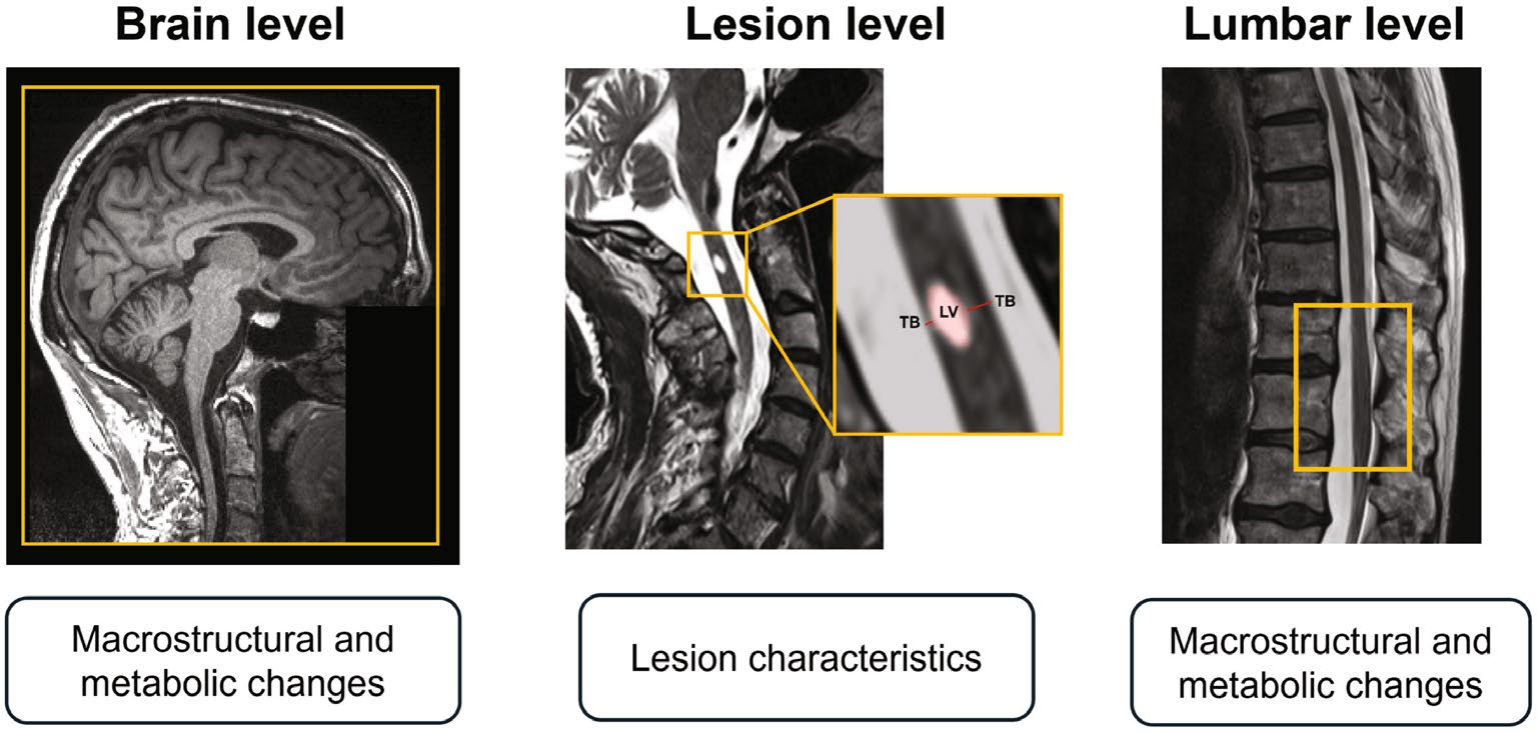
MR acquisition across the motor system. Example of images acquired on an SCI patient across the motor system, including a 3D T1‐weighted acquisition of the brain, a sagittal T2‐weighted MRI of the lesion level with representation of the lesion parameters (lesion volume (LV) and tissue bridges (TB)), and a sagittal T2‐weighted MRI of the lumbar level, with the lumbar cord enlargement highlighted.

All MR measurements were performed on a 3 T MRI scanner (Magnetom Prisma, Siemens Healthineers, Erlangen, Germany), using a standard Siemens 64‐channel head and neck receive radiofrequency (RF) coil for the brain, while a 32‐channel spine receive coil and an 18‐channel body receive coil were used for covering the lumbar cord. Legs were positioned into a vacuum cushion to reduce motion during the lumbar scan. A sagittal T_2_‐weighted (T_2_‐w) sequence covering the spinal cord lesion was added to the brain or lumbar protocol depending on the lesion level. An example of images acquired in an SCI patient is shown in Figure 1.

#### 2.3.1 Brain Protocol

The brain MR protocol consisted of structural T_1_‐weighted (T_1_‐w) and T_2_‐w MRI sequences, B_0_ shimming, and the single‐voxel MRS sequence of the motor cortex. Total acquisition time was approximately 35 min (anatomical sequences: 21 min; MRS sequences: 14 min). All sequence parameters are reported in Table 1.

**TABLE 1:** Sequence parameters. (A) Parameters used for the anatomical sequences in the brain and lumbar cord. (B) Parameters used for the single‐voxel MRS sequences in the brain and lumbar cord.

Brain	TR [ms]	TE [ms]	Slices (n)	FOV [mm^2^]	Resolution [mm^3^]	Flip angle [°]	Bandwidth [Hz/pixel]
(A) Anatomical sequences in the brain and lumbar cord							
Whole brain sagittal T_2_‐weighted	6000	100	25	220 × 247	0.4 × 0.4 × 4	150	222
Sagittal T_2_‐weighted (leg area)	5000	96	25	256 × 208	0.5 × 0.5 × 1.6	150	222
Transversal T_2_‐weighed (leg area)	5000	96	25	256 × 208	0.5 × 0.5 × 1.6	150	222
Coronal T_2_‐weighted (leg area)	5680	96	30	256 × 208	0.5 × 0.5 × 1.6	150	222
3D T_1_‐weighted	2300	2.32	192	240 × 240	0.9 × 0.9 × 0.9	8	200
**Lumbar cord**	**TR [ms]**	**TE [ms]**	**Slices (n)**	**FOV [mm** ^**2**^ **]**	**Resolution [mm** ^**3**^ **]**	**Flip angle [°]**	**Bandwidth [Hz/pixel]**
Sagittal T_2_‐weighted	3000	89	15	330 × 330	0.7 × 0.7 × 4	154	272
Transversal T_2_‐weighed	3000	82	15	190 × 190	0.6 × 0.6 × 4	160	252
Coronal T_2_‐weighted	3000	76	15	260 × 260	0.7 × 0.7 × 4	154	250
Transversal T_2_*‐weighted	38	[6.85, 10.85, 14.85, 18.85, 22.85]	16	192 × 192	0.5 × 0.5 × 5	8	260

	TR [ms]	TE [ms]	Voxel size [mm^3^]	Number of shots	Bandwidth [Hz]
(B) Single‐voxel MRS sequences in the brain and lumbar cord					
Brain	2500	35	20 × 10 × 14	256 (128/run)	2000
Lumbar cord	2000	41	6 × 8 × 35	512 (256/run)	2000

Abbreviation: MRS, magnetic resonance spectroscopy.

**FIGURE 2:**
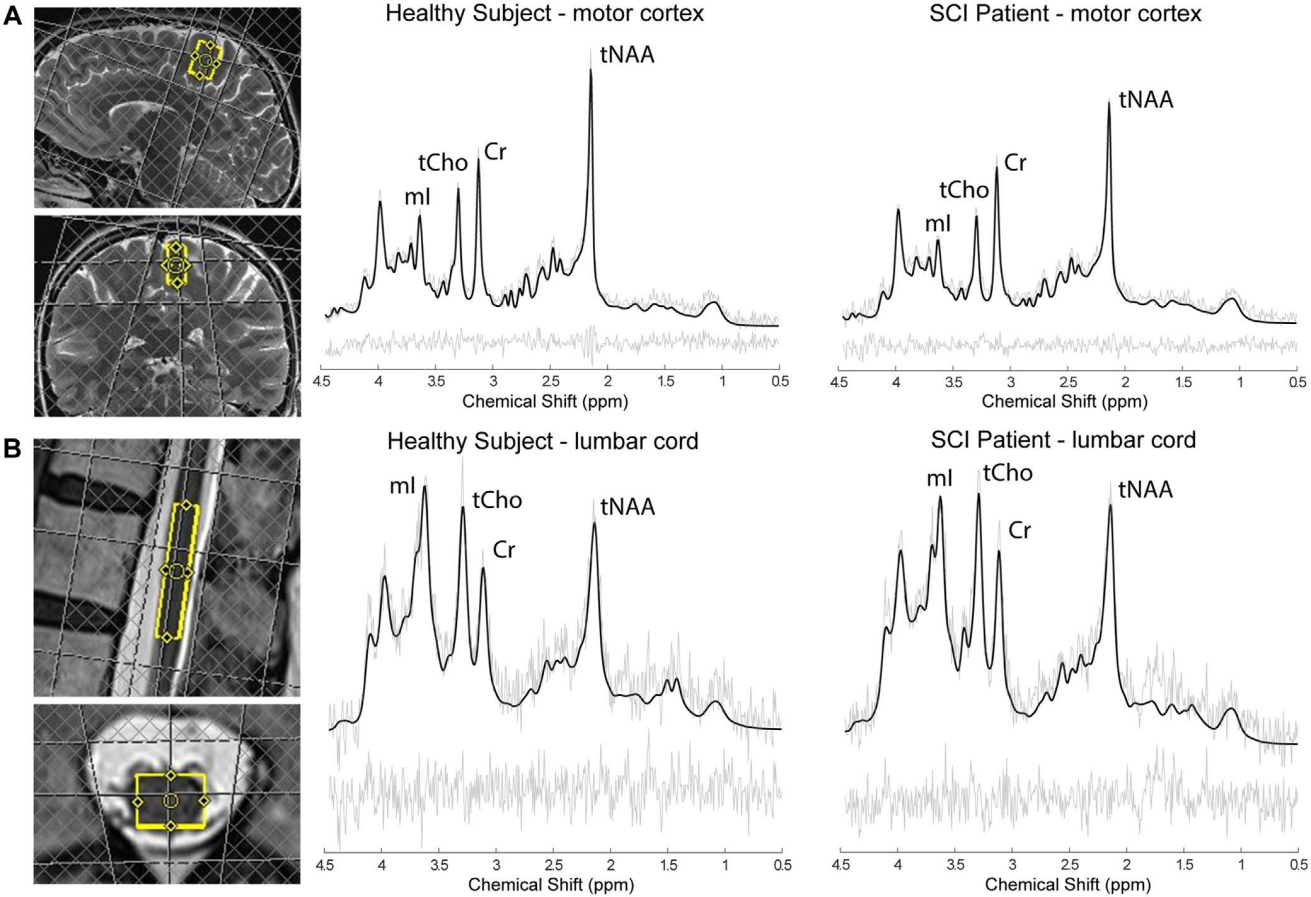
Representative spectra and voxel placement in (A) the motor cortex and (B) the lumbar cord enlargement of a healthy subject and patient with SCI. Black line shows the resulting fit and gray lines show the original spectra and the residuals.

First, structural Turbo Spin Echo (TSE) T_2_‐w images were acquired (Seif et al. 2019) as reference for placing the MRS voxel. Prior to MRS acquisition, B_0_ shimming (first and second order shims) was performed using FASTESTMAP (Gruetter and Tkáč 2000). The MRS voxel (20 × 10 × 14 mm^3^) was placed in the leg area of the primary motor cortex on the more clinically impaired side of SCI patients (Figure 2A). The MRS sequence consisted of a custom‐made semi‐LASER sequence (Scheenen et al. 2007) combined with metabolite cycling (Döring et al. 2018), with a TR of 2500 ms, TE of 35 ms, and no triggering. The semi‐LASER sequence used an optimized symmetric excitation pulse with 8.72 kHz bandwidth and typically 1.9 ms duration. Hyperbolic secant adiabatic refocusing pulses were used with 25.6 kHz bandwidth and 5.5 ms length. Resulting inter‐pulse times (center to center, and center of last pulse to echo maximum) were 4.7, 7.9, 6.7, 9.6, and 6.9 ms. In total, 256 individual shots were recorded across two separate runs of 128 each. Eight unsuppressed water echoes (TEs = 35, 1000, 50, 400, 200, 75, 100, 140 ms, TR = 6000 ms) were acquired to determine the parenchymal vs. cerebrospinal fluid (CSF) water signal for absolute quantification of total Creatine (tCr = Creatine (Cr) + phosphocreatine (PCr)). For structural assessment of the cortex, high‐resolution 3D T_1_‐w images covering the entire brain were acquired.

#### 2.3.2 Lumbar Cord Protocol

The lumbar cord MR protocol included structural T_2_‐w and T_2_*‐w sequences (Seif et al. 2019), B_0_ shimming, and single‐voxel MRS. The total acquisition time was approximately 34 min (anatomical sequences: 7 min; MRS sequences: 27 min), although in some cases, the shimming procedure was repeated to improve spectral quality, which could extend the total time by up to 5 additional minutes. Structural T_2_‐w images of the LCE were acquired in the sagittal, coronal, and transversal planes to position the MRS voxel. Then, B_0_ shimming was conducted as in the brain protocol. The MRS voxel (6 × 8 × 35 mm^3^) was positioned in the LCE (Figure 2B), using the untriggered semi‐LASER sequence combined with metabolite cycling, with a TR set to 2000 ms and TE to 41 ms. In one case, the TR had to be extended to 3000 ms to remain within the SAR specifications. The sequence used an optimized symmetric excitation pulse with 8.72 kHz bandwidth and typically 3.4 ms duration. Hyperbolic secant adiabatic refocusing pulses were used with 25.6 kHz bandwidth and 4.5 ms length. Resulting inter‐pulse times were 4.95, 6.9, 5.7, 13.6, 9.85 ms (center to center, and center of last pulse to echo maximum). Water‐selective inversion recovery was used to suppress the signal from the CSF to properly motion‐filter and phase‐ and frequency‐correct the signal for the metabolites based on the water signal from parenchymal tissue only. In total, 512 shots were recorded from two separate runs of 256 each. Eight unsuppressed water echoes (TEs = 41, 1000, 50, 400, 200, 75, 100, 140 ms, TR = 6000 ms) were acquired to determine the absolute tCr concentration as in the brain. To assess volumetric changes, a transversal 3D T_2_*‐w sequence was acquired covering the LCE.

### 2.4 MRS Processing

For both the brain and LCE, individual metabolite‐cycled non‐water suppressed echoes were phase‐, frequency‐, and eddy‐current corrected. Motion‐related artifacts were removed using a motion compensation (MoCom) scheme in Matlab 2023b (MathWorks, Natick, MA, USA), detecting outlying single shots based on the distribution of frequency, amplitude, and linewidth for the water signals in the individual single shots of the metabolite cycling acquisition (Döring et al. 2018). Exclusion of low‐quality single shots was done if the relative fitting uncertainty of the estimated amplitude or linewidth exceeded 5%. Next, individual echoes were combined according to the metabolite cycling scheme. The resulting water‐suppressed average spectrum was phase‐corrected, and the remaining spurious water signal was removed by singular value decomposition using the Lanczos algorithm in jMRUI (v7.01) (Naressi et al. 2001; Stefan et al. 2009). Then, echoes of both runs were averaged to increase the signal‐to‐noise ratio (SNR) and improve model fitting. Quantification of individual metabolites was achieved by linear combination model fitting using FitAID (Adalid et al. 2017; Chong et al. 2011) with the following metabolites in the basis set: aspartate, creatine, γ‐aminobutyric acid (GABA), glucose, glutamate (Glu), glutamine (Gln), glutathione, glycine, glycerophosphorylcholine (GPC), lactate, myo‐inositol (mI), N‐acetylaspartate (NAA), N‐acetylaspartylglutamate (NAAG), phosphocreatine (PCr), phosphorylcholine (PCho), phosphorylethanolamine, scyllo‐inositol, taurine, and a macromolecular background signal (MMBG). This MMBG was estimated based on a cohort average of the healthy controls by modeling an overall MMBG as a set of equally spaced Voigt lines (5 Hz spacing, 14 Hz Lorentz width, 7.8 Hz Gauss width for the brain and 0.7 Hz Gauss width for the spinal cord) in addition to the metabolites' basis spectra.

The individual non‐water‐suppressed acquisitions were eddy‐current corrected, and the signal decay was modeled using a two‐compartment model in FitAID to determine the parenchymal vs. CSF water signal contribution (Ernst et al. 1993). Using the parenchymal water signal and the metabolite‐specific T_1_ and T_2_ relaxation times (Träber et al. 2004), the metabolite signal intensities of tCr were converted into millimolar concentrations for absolute quantification with the following equation (Pfyffer et al. 2023; Near et al. 2021):

$${\left[M\right]}_{\text{molar}}=\frac{S_{M}}{S_{H_{2}O}}\cdot \frac{1}{1-\exp\left(-\frac{T_{R}}{T_{1}}\right)}\cdot \frac{1}{\exp\left(-\frac{T_{E}}{1.5\cdot T_{2}}\right)}\cdot \left(f_{\mathrm{GM}}d_{\mathrm{GM}}+f_{\mathrm{WM}}d_{\mathrm{WM}}\right){\left[H_{2}O\right]}_{\text{molar}}$$

Where *S*_*M*_ and *S*_H20_ refer to the metabolite and parenchymal water signal intensities respectively, including the scaling factors for the number of protons per molecule. T_2_ values were multiplied by a factor of 1.5 to account for the slower T_2_ relaxation in semi‐LASER sequences (Allaïli et al. 2015; Deelchand et al. 2015). *f*_GM_ and *f*_WM_ represent the volume fractions of gray and white matter within the MRS voxel as calculated from tissue segmentations (excluding the CSF compartment) based on the T_1_‐w MPRAGE images using SPM12 (version 6906, University College London, London, UK) for the brain and based on manual segmentations of the T_2_*‐w image using JIM (version 9, Xinapse Systems, Aldwincle, UK) for the spinal cord. Volume fractions amounted to 0.38 for GM and 0.62 for WM in the brain, and to 0.36 and 0.64 in the lumbar cord. *d*_GM_ and *d*_WM_ refer to the GM‐ and WM‐specific water content as reported in literature (GM: 0.78, WM: 0.65) (Ernst et al. 1993) and [H_2_O]_molar_ refers to the molar concentration of water (Near et al. 2021).

Due to incorrect setting of the echo times of the water echo series in some participants and exceeding SAR limit requiring adjustment of the reference amplitude during the scan, absolute tCr concentrations were estimated only in a subset of the entire dataset (brain: 11 HC/8 SCI patients, spinal cord: 18 HC/11 SCI patients) and compared between SCI and HC using two‐sided Welch's *t*‐tests to assure no systematic differences in tCr concentration between both groups. Relative concentrations of the metabolites of interest (total N‐acetylaspartate (tNAA = NAA + NAAG), total choline‐containing compounds (tCho = GPC + PCho), and myo‐inositol (mI)), normalized to tCr, were analyzed across the full dataset.

#### 2.4.1 Spectral Quality

Spectra of low quality were excluded based on the Gauss linewidth, as a measure of shim quality, and the Cramér‐Rao lower bound (CRLB), which represents the minimal error of metabolite fitting (Near et al. 2021; Kreis 2015). To prevent bias from exclusion criteria based on percentage values and disparate group composition for different metabolite comparisons, whole spectra, rather than single metabolite estimates, were excluded based on absolute CRLBs (i.e., in mM units). It is important to mention that the metabolites Cr and PCr, as well as GPC and PCho, are likely to be highly correlated at 3 T MRI (Ju et al. 2024; Bell et al. 2025). However, this study reports here the CRLBs of metabolites individually since the fitting program (FitAID (Adalid et al. 2017; Chong et al. 2011)) does not provide summed values for metabolites directly, nor error bounds for the sums. The specific CRLB‐related exclusion criteria were defined as exclusion of a spectrum if CRLBs of more than half of the analyzed metabolites exceeded a cutoff value defined by 1.5 times or 2.0 times their median cohort value, in the brain and lumbar cord respectively (slightly more stringent for the brain than the lumbar cord, because of the generally higher spectral quality in the brain). In addition, spectra were excluded if the common metabolite Gaussian linewidth in the model fitting was larger than 7.5 Hz (Pfyffer et al. 2023) (in the brain) or 10 Hz (in the lumbar cord).

### 2.5 MRI Processing

#### 2.5.1 Structural Brain Data

Voxel‐based morphometry (VBM) analysis was conducted in SPM12 (v6906; University College London, London, UK) using the structural 3D T_1_‐w MPRAGE images. Images were automatically segmented into different tissue classes (including GM, WM, and cerebrospinal fluid) using unified segmentation (Ashburner and Friston 2005). Probabilistic tissue maps were normalized into the standard Montreal Neurological Institute (MNI) space using the DARTEL algorithm (Ashburner 2007) and smoothed using an isotropic 6 mm FWHM Gaussian kernel.

#### 2.5.2 Structural Lumbar Cord Data

Cross‐sectional areas of white matter (WMA) and gray matter (GMA) were obtained on the transversal T_2_*‐w images by conducting manual segmentations in JIM. Values were averaged over three slices centered on the slice with the largest spinal cord area (Büeler et al. 2024).

#### 2.5.3 Lesion Characterization

Lesion volumes of patients were obtained from the sagittal T_2_‐w MRI using the *sct_deepseg* algorithm (*seg_sc_lesion_t2w_sci* task, v6.2) (Karthik et al. 2024) to generate lesion masks, which were manually corrected if necessary, and *sct_analyze_lesion* to extract volume values. Preserved anterior and posterior tissue bridges were derived manually in JIM as described previously (Huber et al. 2017) (Figure 1).

### 2.6 Statistical Analyses

All statistical analyses except for the VBM analysis were conducted using R (v4.3.01) in RStudio (v2023.06.1).

#### 2.6.1 Metabolite Analysis

To assess whether MRS data quality was similar between the two cohorts, CRLBs and Gauss linewidths were compared between HC and SCI patients using two‐sided Welch's *t*‐tests. Metabolite concentration ratios were analyzed using Welch's *t*‐tests (*α* = 0.05), focusing on decreased tNAA (i.e., less viable and dense neurons), decreased tCho (i.e., less intact membranes), and increased mI (i.e., activated glial cells indicative for gliosis) based on prior findings (David, Mohammadi, et al. 2019; Wyss et al. 2019; David et al. 2021; Pfyffer et al. 2020).

Associations between metabolite concentrations in the LCE and brain were explored using linear models. In SCI patients, Spearman and Pearson correlations assessed relationships between lumbar metabolite concentrations and lesion level, lesion characteristics (lesion volume, area, width, length and tissue bridges), and functional outcomes (lower extremity motor score, lower extremity light‐touch and pin‐prick [dermatomes L2—S2]), as well as time since injury. Additionally, associations between brain metabolite concentrations and lesion characteristics were assessed using Pearson correlations.

#### 2.6.2 Structural Analysis

Volumetric differences in the motor cortex between SCI and HC were assessed using a general linear model in SPM12, accounting for age and sex as potential confounding factors. Voxel‐wise one‐sided two‐sample t‐tests within the bilateral motor cortex assessed volumetric decreases, with a significance threshold of *p* < 0.05 (family‐wise error‐corrected). To enhance sensitivity to changes within the leg area of the primary sensory and motor cortex, a 10‐mm sphere was centered on x =−4 mm, y =−46 mm, and z = 62 mm, and x =−6 mm, y =−28 mm, z = 60 mm, respectively, following prior reports (Freund, Weiskopf, et al. 2011).

At the lumbar level, WMA and GMA differences between SCI and HC were compared using a two‐sample Welch's *t*‐test (*α* = 0.05). Due to the presence of motion and/or susceptibility artifacts in the lumbar cord, the comparison of WMA and GMA was conducted on a subset of the whole cohort (HC: 15 subjects, SCI: 11 subjects).

## 3 Results

### 3.1 Demographics and Clinical Characteristics

SCI patients and HC did not differ with respect to age (*p* = 0.80, Welch's *t*‐test) and sex (*p* = 0.07, Fisher's exact test). The average time since injury was 7.9 ± 8.2 years (range 0.58–24.3 years). The (neurological) level of injury ranged from C1 to T12. Two patients with SCI were classified based on the American Spinal Injury Association Impairment Scale (AIS) as AIS A and 14 as AIS D. Clinical and neurological characteristics are reported for the SCI cohort in Table 2.

**TABLE 2:** Clinical characteristics across the SCI cohort.

Age [years] (mean ± SD)	54.7 ± 14.8
Sex	14 m, 2 f
Time since injury [years] (mean ± SD)	7.9 ± 8.2
Neurological level of injury	C1 (*n* = 2), C3 (*n* = 3), C4 (*n* = 1), C5 (*n* = 2), C6 (*n* = 1), T1 (*n* = 1), T2 (*n* = 1), T3 (*n* = 1), T4 (*n* = 2), T11 (*n* = 1), T12 (*n* = 1).
AIS score	A (*n* = 2), D (*n* = 14)
Lesion volume [mm^3^] (mean ± SD [min–max])	128.18 ± 175.45 [0–602.13]
Tissue bridges [mm] (mean ± SD [min–max])	3.42 ± 2.22 [0–7.18]
LEMS (median [min–max])	44 [0–49]
LELT (median [min–max])	12 [0–24]
LEPP (median [min–max])	11 [0–24]

Abbreviations: LELT, lower extremity light‐touch (dermatomes L2–S2); LEMS, lower extremity motor score; LEPP, lower extremity pin‐prick (dermatomes L2–S2).

### 3.2 Quality of MR Spectroscopy Measurements

The analysis included 18 HC and 15 SCI for the brain and 19 HC and 15 SCI for the lumbar cord. Representative spectra of a HC and an SCI patient from the motor cortex and the spinal cord are shown in Figure 2. The brain scan for one HC was interrupted due to a scanner malfunction. In one SCI patient, cervical spine fixation prevented proper positioning within the RF head coil. Additionally, lumbar cord data was not acquired for another SCI patient.

On average, 17.6% of the individual transients were excluded using the MoCom scheme (Döring et al. 2018) in the brain of HC and 21.51% in SCI. In the lumbar cord, 10.8% and 8.0% of the echoes were excluded in HC and SCI, respectively.

The average Gauss linewidth in the brain was 3.4 ± 0.7 Hz in HC and 3.6 ± 0.5 Hz in SCI patients. In the LCE, the linewidths were 6.4 ± 1.3 Hz and 6.7 ± 1.2 Hz in HC and patients, respectively. No significant differences were observed in the linewidths between patients with SCI and HC in the brain or lumbar cord.

**FIGURE 3:**
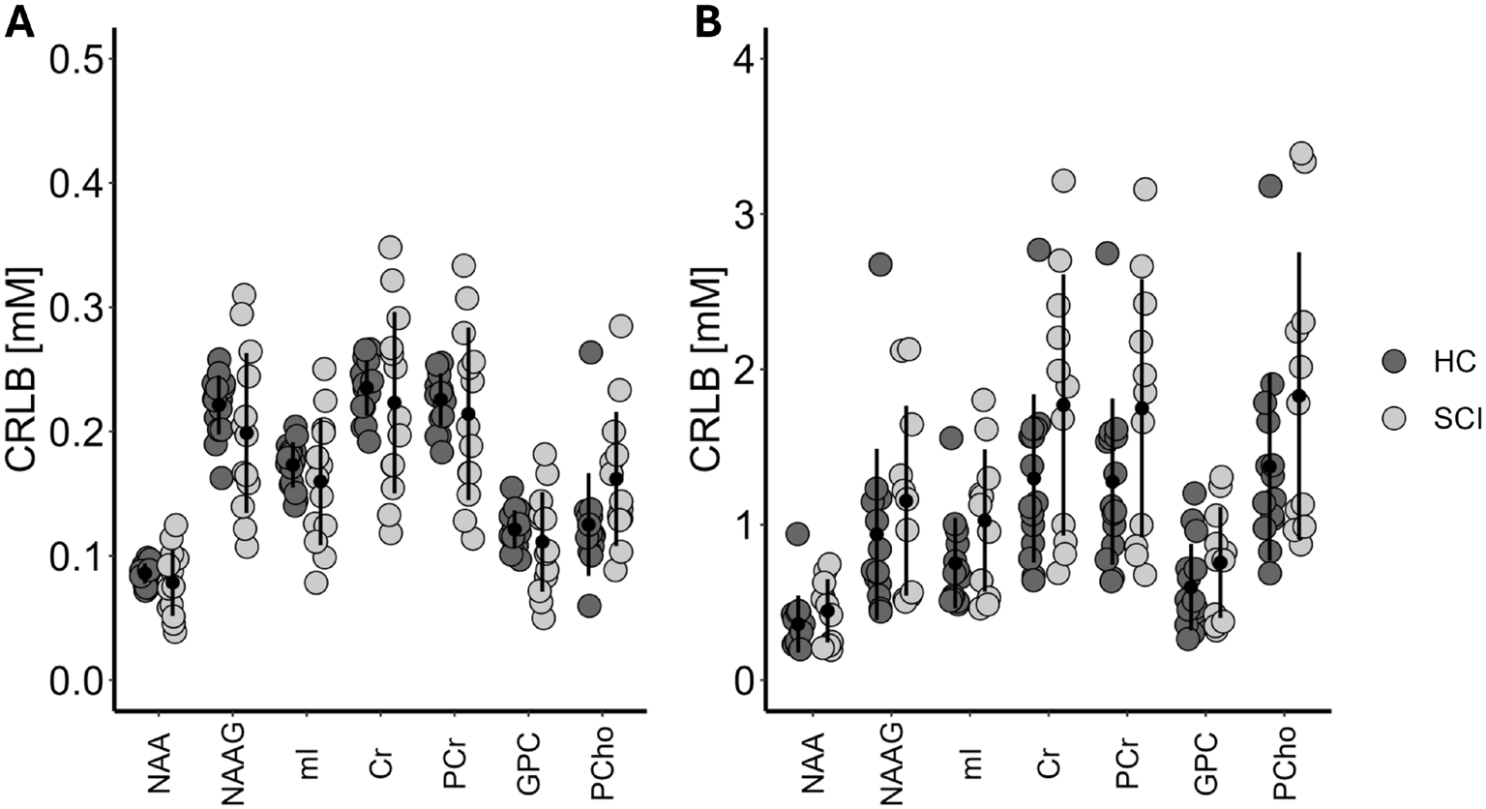
Cramér‐Rao lower bound (CRLB) distributions of the different metabolites in the (A) primary motor cortex and (B) lumbar cord enlargement for healthy controls (HC) and patients with SCI (SCI). Cr, creatine; GPC, glycerophosphorylcholine; mI, myo‐inositol; NAA, N‐acetyl aspartate; NAAG, N‐acetylaspartylglutamate; PCho, phosphorylcholine; PCr, phosphocreatine.

In the brain, the mean CRLB for each metabolite of interest separately was below 0.5 mM, after exclusion of poor‐quality spectra, while in the lumbar cord it was below 1.83 mM, across both cohorts. There were no significant differences in the CRLBs between subjects with SCI and HC for each of the metabolites in the brain, nor in the lumbar cord. Based on the exclusion criteria for the CRLB and Gauss linewidth, four subjects had to be excluded from the brain analysis (2 HC, 2 SCI) and eight from the lumbar cord analysis (4 HC, 4 SCI). Figure 3 shows the individual CRLBs for every metabolite analyzed in this study for patients with SCI and HC separately.

### 3.3 Metabolite Concentrations in the Brain

**FIGURE 4:**
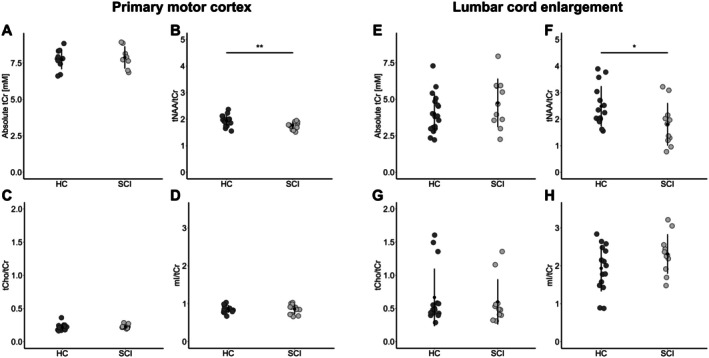
Metabolite changes across the motor system. The dotplots show the metabolite concentration (A, E) and metabolite ratios (B–D, F–H) in the brain primary motor cortex and lumbar cord enlargement of healthy controls (HC) and patients with spinal cord injury (SCI). mI, myo‐inositol; tCho, total choline‐containing compounds; tCr, total creatine; tNAA, total N‐acetyl aspartate. *p < 0.05. **p < 0.01.

There was no significant difference in absolute tCr concentration comparing patients with SCI and HC in the primary motor cortex (HC: 7.74 ± 0.67 mM, SCI: 7.88 ± 0.76 mM, *t*(17) = −0.42, *p* = 0.68) (Figure 4A).

Regarding the metabolites of interest, we found a significantly lower ratio of tNAA/tCr in SCI patients compared to HC (HC: 1.94 ± 0.21, SCI: 1.77 ± 0.14, ∆ = −8.8%, *t*(27) = 2.71, *p* = 0.006) (Figure 4B). In contrast, there was no evidence for lower tCho/tCr (HC: 0.22 ± 0.05, SCI: 0.23 ± 0.03, *t*(27) = −0.75, *p* = 0.77) or higher mI/tCr (HC: 0.87 ± 0.10, SCI: 0.86 ± 0.13, *t*(27) = 0.13, *p* = 0.55) in SCI (Figure 4C,D).

### 3.4 Metabolite Concentrations in the LCE

There was no significant difference in absolute tCr concentration between patients with SCI and HC in the LCE (HC: 4.02 ± 1.33 mM, SCI: 4.75 ± 1.66 mM, *t*(27) = −1.24, *p* = 0.23) (Figure 4E). We observed a significant decrease in the tNAA/tCr ratio in SCI compared to HC (HC: 2.48 ± 0.76, SCI: 1.81 ± 0.80, ∆ = −27.0%, *t*(24) = 2.17, *p* = 0.02) (Figure 4F). Patients with SCI did not exhibit lower tCho/tCr (HC: 0.67 ± 0.43, SCI: 0.60 ± 0.34, *t*(24) = 0.44, *p* = 0.33), but there was a trend toward higher mI/tCr (HC: 1.94 ± 0.61, SCI: 2.31 ± 0.52, *t*(24) = −1.71, *p* = 0.051) compared to HC (Figure 4G,H).

### 3.5 Volumetric/Atrophy Analysis

**FIGURE 5:**
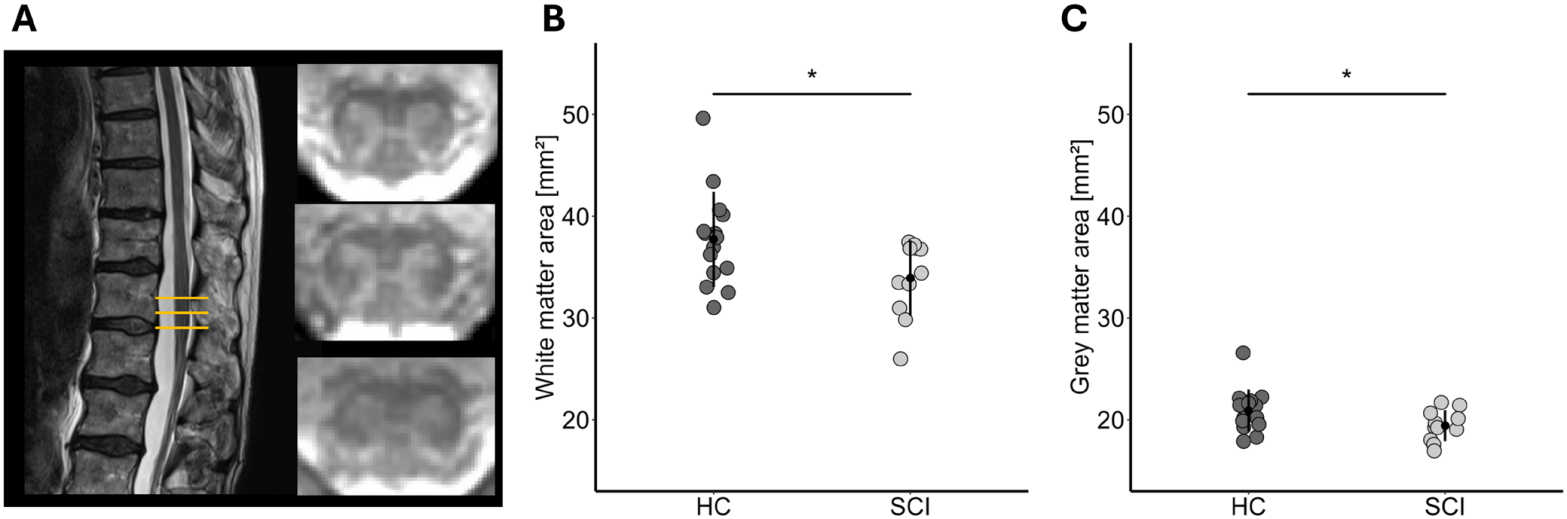
Tissue‐specific atrophy in the lumbar cord enlargement. (A) Representative example of the anatomical sagittal T_2_‐ and axial T_2_*‐weighted images. Decrease of the cross‐sectional area of the (B) white matter and (C) gray matter in healthy controls (HC) and patients with SCI (SCI). *p < 0.05.

We found a decreased WMA in patients with SCI compared to HC (HC: 37.7 ± 4.7 mm^2^, SCI: 33.9 ± 3.7 mm^2^, Δ = −10.1%, *t*(24) = 2.32, *p* = 0.015) (Figure 5B). Likewise, the GMA was significantly lower in patients with SCI (HC: 20.9 ± 2.1 mm^2^, SCI: 19.4 ± 1.5 mm^2^, Δ = −7.2%, *t*(24) = 2.12, *p* = 0.022) (Figure 5C).

In the brain, a trend was observed in the GM volume of the leg area of the sensory cortex (z‐score = 3.26, x = −8 mm, y = −44 mm, z = 70 mm, *p* = 0.054, corrected for multiple comparisons). There were no significant differences in the GM volume of the leg area of the motor cortex when comparing patients with SCI and HC.

### 3.6 Associations With Clinical Outcomes

No significant associations were observed between metabolite changes in the LCE and functional outcomes, lesion characteristics, or time since injury in patients with SCI. Furthermore, metabolite changes in the LCE did not correlate with changes in the primary motor cortex. Finally, there were no significant correlations between the brain metabolite concentrations and lesion characteristics.

## 4 Discussion

This study showed significant metabolic changes in SCI patients indicating neuronal atrophy in both the primary motor cortex (i.e., retrograde neurodegeneration) and the LCE (i.e., trans‐synaptic neurodegeneration).

Our findings revealed a decrease in markers of neuronal integrity and density in both the leg area of the primary motor cortex and in the LCE, suggesting SCI‐induced metabolic changes in rather mildly impaired SCI compared to HC using ^1^H‐MRS. In parallel, atrophy of LCE, characterized by reductions in both WM and GM cross‐sectional cord areas, was detected, consistent with previous findings (David, Seif, et al. 2019; David et al. 2022). Thus, profound pathophysiological alterations occur in the motor system across the CNS rostro‐caudal to the injury and can be detected on various scales (i.e., metabolic environment and macrostructure). Crucially, metabolic markers were able to detect subtle signs of remote neurodegeneration in the brain, pointing at their potential for detecting changes early after injury.

### 4.1 Changes in the Motor Cortex

Animal models suggest that primary motor neurons in the motor cortex degenerate and shrink following lesions of the corticospinal tract resulting from retrograde neurodegeneration (Kalil and Schneider 1975; Barron et al. 1988). In human SCI, injury‐induced macrostructural and microstructural degeneration, including demyelination and axonal loss, were reported in the motor cortex and the corticospinal tracts (David, Seif, et al. 2019; Azzarito et al. 2020; Emmenegger et al. 2024; Schading et al. 2023). Using ^1^H‐MRS, we found reductions in tNAA as a marker for neuronal density and integrity in the motor cortex of SCI patients. This suggests neuronal shrinkage occurring in human SCI because of retrograde neurodegeneration and provides in vivo evidence for neurodegenerative processes, in line with a previous MRI study (Liu et al. 2023). However, there was no change in markers of membrane integrity or glial activation. This could be explained in chronic SCI patients due to tCho levels decreasing over time following the initial phase of active membrane turnover after injury caused by cell breakdown or demyelination (Qian et al. 2010). SCI is associated with an active glial reaction near the lesion (Silver and Miller 2004), but no extensive glial activation was detected around motor neurons in the motor cortex, in line with the absence of an increase in mI (Leong et al. 1995; Shokouhi et al. 2010). Despite evidence of neuronal shrinkage, VBM showed no macrostructural changes in the motor cortex of the leg area. While the sensory cortex was not the primary focus of this study, a trend toward reduced sensory cortex volume was observed in SCI, likely reflecting the mild‐to‐moderate impaired chronic AIS D patients, who retained walking ability. This suggests that metabolic markers for changes in microstructure may be sensitive to detect subtle changes and to monitor treatment effects, as shown in different pathologies such as multiple sclerosis and gliomas (John et al. 2023; van Dijken et al. 2017).

### 4.2 Changes in the LCE

Animal models showed trans‐synaptic degeneration from reduced synaptic input of the preceding neuron (Ginsberg and Martin 2002) and electrophysiological changes of the secondary motor neurons in the LCE following SCI (Chang 1998; Dietz 2004). MRI studies have shown extensive GM and WM atrophy along with microstructural changes, indicative of axonal degeneration, demyelination, and neuronal cell body changes (David, Seif, et al. 2019). Our findings confirm these macrostructural changes with reductions in both WMA and GMA.

Moreover, ^1^H‐MRS results showed significantly reduced tNAA levels in the LCE, indicating reduced neuronal integrity or density due to trans‐synaptic neurodegeneration of secondary motor neurons below the injury. However, unchanged tCho levels between SCI and HC suggested no altered membrane turnover in the LCE in chronic SCI. Finally, while ml levels were not significantly different between chronic SCI patients and HC, there was a trend toward an increase in SCI, which may suggest reactive gliosis in the LCE (David, Mohammadi, et al. 2019; Soares and Law 2009; Silver and Miller 2004).

Notably, tNAA reduction was more pronounced in the LCE (−27.0%) than in the primary motor cortex (−8.8%).

This study has several limitations. The small, heterogeneous SCI cohort, with mostly mild‐to‐moderate lower limb deficits and predominantly AIS D rated, may have limited statistical power and sensitivity to metabolic changes. However, this limitation is common across many SCI studies, as the condition is inherently heterogeneous, and subject recruitment is often challenging. Previous studies, such as from Freund et al. (Freund, Rothwell, et al. 2011; Freund et al. 2013), Grabher et al. (Grabher et al. 2015), and David et al. (David, Seif, et al. 2019), also included similar sample sizes. Furthermore, acquisition in the LCE faces inherent challenges, including more pronounced B_0_ field distortions due to susceptibility changes caused by surrounding tissues, vicinity to the lungs, the distance from the receive coils, and the influence of respiration and cardiac pulsation (Hock et al. 2012). However, recent advances in MRS technical development have enabled its acquisition and clinical application of MRS in the lumbar cord, such as the semi‐LASER MRS approach used in this study, which is relatively insensitive to field inhomogeneities (Scheenen et al. 2007). Despite lower spectral quality in the LCE compared to the brain, with higher CRLB values in both groups, our results still allowed for meaningful metabolite comparisons between SCI and HC.

## 5 Conclusion

Our study demonstrates significant metabolic changes in SCI patients, indicating neuronal atrophy both in the primary motor cortex (reflecting retrograde neurodegeneration) and the LCE (reflecting trans‐synaptic neurodegeneration). These findings provide in vivo evidence of remote neuronal changes due to secondary neurodegenerative processes along the neuraxis. Given the lumbar region's key role in lower limb function, the feasibility of lumbar cord MRS represents a technical achievement, with great potential to assess the integrity of the LCE and provide valuable insights into neurodegenerative processes. Ultimately, it holds potential for developing early biomarkers to improve treatment strategies in the future and monitor subtle changes following SCI.

## Author Contributions

**Simon Schading‐Sassenhausen:** investigation, formal analysis, writing – original draft, visualization. **Anna Lebret:** investigation, formal analysis, writing – original draft, visualization. **Kadir Şimşek:** software, writing – review and editing. **Pauline Gut:** investigation, writing – review and editing. **Sabrina Imhof:** resources, writing – review and editing. **Björn Zörner:** conceptualization, funding acquisition, writing – review and editing. **Roland Kreis:** software, methodology, writing – review and editing. **Patrick Freund:** conceptualization, funding acquisition, writing – review and editing. **Maryam Seif:** conceptualization, methodology, investigation, funding acquisition, writing – review and editing.

## Conflicts of Interest

The authors declare no conflicts of interest.

## Supporting information


Data S1.

## Data Availability

The data that support the findings of this study are available upon reasonable request from the corresponding author.
